# The effects of acute aerobic exercise on appetite‐regulating parameters and energy intake in males with obesity

**DOI:** 10.1002/hsr2.70067

**Published:** 2024-09-10

**Authors:** Shahin Khodabandeh, Farhad Rahmani‐nia, Bahman Mirzaei, Timothy J. Fairchild, Tom J. Hazell

**Affiliations:** ^1^ Department of Exercise Physiology University of Guilan Rasht Iran; ^2^ School of Allied Health and Centre for Healthy Ageing College of Health and Education, Murdoch University Murdoch Western Australia Australia; ^3^ Department of Kinesiology and Physical Education Wilfrid Laurier University Waterloo Canada

**Keywords:** anorexigenic, appetite regulation, energy intake, obesity, orexigenic

## Abstract

**Objective:**

To investigate the effects of moderate‐intensity aerobic exercise on appetite control parameters, appetite perceptions, and energy intake in sedentary males with obesity.

**Design:**

Eleven males with obesity (body fat percentage 36.5 ± 2.5%, body mass index 35.3 ± 4.2 kg/m^2^, V̇O_2peak_ 29 ± 3.1 mL·kg^−1^·min^−1^) completed two experimental sessions: (1) no exercise (CTRL) and (2) 60 min of moderate‐intensity cycling exercise at 60% V̇O_2peak_ (MICT) in a crossover design. Blood analysis included growth differentiation factor 15 (GDF‐15), total ghrelin, peptide tyrosine tyrosine_3–36_ (PYY_3–36_), total glucagon‐like peptide‐1 (GLP‐1), insulin, and glucose, as well as subjective appetite perceptions were measured in specific intervals. A standard breakfast at 0 h and an ad libitum meal postexercise was provided.

**Result:**

GDF‐15 (95% confidence interval [CI]: [2.48–27.28] ng/L, *p* = 0.021) increased immediately following MICT compared to CTRL. However, there were no differences for PYY_3–36_ (*p* = 0.480, $\eta_{p}^{2}=0.025$), total ghrelin (*p* = 0.646, $\eta_{p}^{2}=0.011$), and total GLP‐1 (*p* = 0.451, $\eta_{p}^{2}=0.029$) between sessions. Appetite perceptions (95% CI: [(−20.38)–(−6.16)] mm, *p* = 0.001) were suppressed following MICT though energy intake was not different between the sessions (95% CI: [(−1904.9)–928.1] kJ, *p* = 0.480).

**Conclusion:**

Sixty minutes of MICT increased GDF‐15 while suppressing appetite perceptions in individuals with obesity. There was no energy compensation postexercise.

## 1 INTRODUCTION

Obesity is defined as the excessive accumulation of fat and is associated with the development of health issues such as diabetes, cardiovascular diseases, and some types of cancers.^1^ According to the World Health Organization, the number of people living with obesity increased to 1 billion in 2022.^2^ In view of the increased global prevalence and negative health outcomes of obesity, there is a concerted effort to improve both preventative measures and the management of obesity. As such, researchers have increasingly focused on the two components of the energy balance equation, namely energy intake and energy expenditure, as well as their interaction.^3^ Specifically, whether an increase in energy expenditure in the form of exercise stimulates appetite and drives energy intake, remains a topic of interest.

Appetite is regulated in the central nervous system which receives input from multiple psychological, environmental, and physiological factors.^4^ Physiological factors include orexigenic (appetite‐stimulating) and anorexigenic (appetite‐inhibiting) factors which can be measured peripherally and include the primary orexigenic peptide ghrelin, which originates from the stomach.^4^ The key anorexigenic peptides include peptide tyrosine tyrosine (PYY) and glucagon‐like peptide‐1 (GLP‐1), where the active forms of these hormones are involved in satiety between meals.^5^ However, some results suggest that people with obesity may have different concentrations of appetite‐regulating hormones,^6^ and they might exhibit varied responses to exercise.

Metabolic changes and factors released in response to exercise can also affect appetite, including lactate and the myokine growth differentiation factor 15 (GDF‐15).^7^ Recent evidence suggests an important role for lactate as an anorexigenic metabolite^8^ while GDF‐15, produced by multiple tissues including skeletal muscles, exerts anorexigenic effects by binding to receptors in the area postrema and hindbrain.^9^ While a few studies have reported an acute increase in GDF‐15 concentrations,^10^, ^11^ evidence in the context of obesity remains limited.

Moderate‐intensity aerobic exercise (at 50%–75% V̇O_2peak_) is recognized as a means to manage body weight^12^ by increasing energy expenditure without the compensatory increase in energy intake.^13^ However, investigations into the effects of aerobic exercise on appetite hormones in overweight and obese individuals yield contradictory results, possibly due to the inclusion of both overweight and obese participants. Dorling and colleagues highlighted the potential impact of body fat on the response of appetite hormones.^13^ Notably, body fat, acting as an independent factor, holds the capacity to modulate appetite, appetite‐related hormone concentrations, and postexercise food intake. This effect is particularly pronounced concerning episodic appetite‐related hormones total ghrelin, PYY, and GLP‐1 where increased body fat correlates with decreased fasting anorexigenic hormones concentrations and diminished postmeal fluctuations.^14^

The appetite‐regulatory responses to acute exercise are not well understood in individuals with obesity. Although body mass index (BMI) is a useful method for evaluating obesity in epidemiological studies, it falls short at the individual level as it does not measure adiposity. Adopting body composition rather than BMI to assess the appetite‐regulatory responses after acute aerobic exercise in sedentary people with obesity is warranted. Individuals with body fat percentages between 32 and 37 are classified as living with class one obesity.^15^ We hypothesized that individuals with higher body fat percentage would exhibit different (reduced suppression) appetite responses following exercise. Therefore, this study will investigate the effects of acute aerobic exercise on appetite‐regulatory factors, subsequent energy intake, and perceptions of appetite in people with obesity and a sedentary lifestyle.

## 2 METHODS

### 2.1 Participants

Participant recruitment occurred following approval from Sport Sciences Research Institute of Iran's Ethics Advisory Committee. All potential participants provided informed consent before assessment of eligibility which included: nonsmoking, body mass stability in the previous 3 months (±2 kg), metabolically healthy, using no medications, living an inactive lifestyle based on the International Physical Activity Questionnaire,^16^ having no abnormal eating behavior (overeating, undereating, and emotional eating),^17^ body fat between 32% and 45%, BMI >30 kg/m^2^, and waist circumference >102 cm. These eligibility criteria were assessed during the first visit (Screening and Familiarization) described below. The participants' characteristics included Age: 22 ± 2 y; BF%: 36.5 ± 2.5; BMI: 35.3 ± 4.2 kg/m^2^; waist circumference: 119.6 ± 7.2 cm; V̇O_2peak_: 28.4 ± 3.4 mL·kg^−1^·min^−1^; low‐density lipoprotein (LDL): 82 ± 17.4 mg.dL; high‐density lipoprotein (HDL): 44.54 ± 3.04 mg/dL; Glucose: 100.45 ± 7.68 mg/dL; total cholesterol: 162.37 ± 30.12 mg/dL; triglycerides: 125.90 ± 37.42 mg/dL; HOMA‐IR: 1.67 ± 0.73.

### 2.2 Screening and familiarization session

To confirm eligibility, participants were asked to arrive at the laboratory at 0800 after a 12‐h overnight fast. Following this, blood samples were collected to assess metabolic health (described below) and height was measured using an electronic measuring scale (Seca), and body fat was measured using the InBody 270 (InBody). To improve the accuracy of bioelectrical impedance measurements, participants were instructed to avoid exercise and refrain from drinking any liquids for 8 h before the body fat measurement. Additionally, waist circumference was measured at the narrowest point of the torso, between the lower rib margin and the iliac crest. Subsequently, a standard breakfast was provided and participants were provided 1 h of rest during which they completed a set of questionnaires to assess health status, habitual physical activity using the International Physical Activity Questionnaire, and psychological eating tendencies with the Three‐Factor Eating Questionnaire.^17^

Participants completed a peak oxygen uptake (V̇O_2peak_) test on a cycle ergometer (Monark LC4, Monark Exercise AB). The test commenced with a 5‐min warm‐up with a resistance of 50 W followed by a gradual increase in the workload by 20 W every 2 min until exhaustion where a pedaling rate between 60 and 70 rpm was required.^18^ Oxygen consumption and carbon dioxide production were measured using an online breath‐by‐breath gas analysis system (Metalyser 3B, Cortex, Biophysik). Heart rate was monitored via telemetry (Polar H10; Polar Electro Oy). The criteria for ensuring attainment of V̇O_2peak_ required participants to meet ≥2 of the following: heart rate within 10 beats min^−1^ of age‐predicted maximum, respiratory exchange ratio ≥1.1, and rating of perceivede exertion ≥19. Participants were given an ad libitum meal after the test to familiarize them with this meal paradigm to minimize over‐eating in the main trial.

### 2.3 Experimental procedure

**Figure 1:**
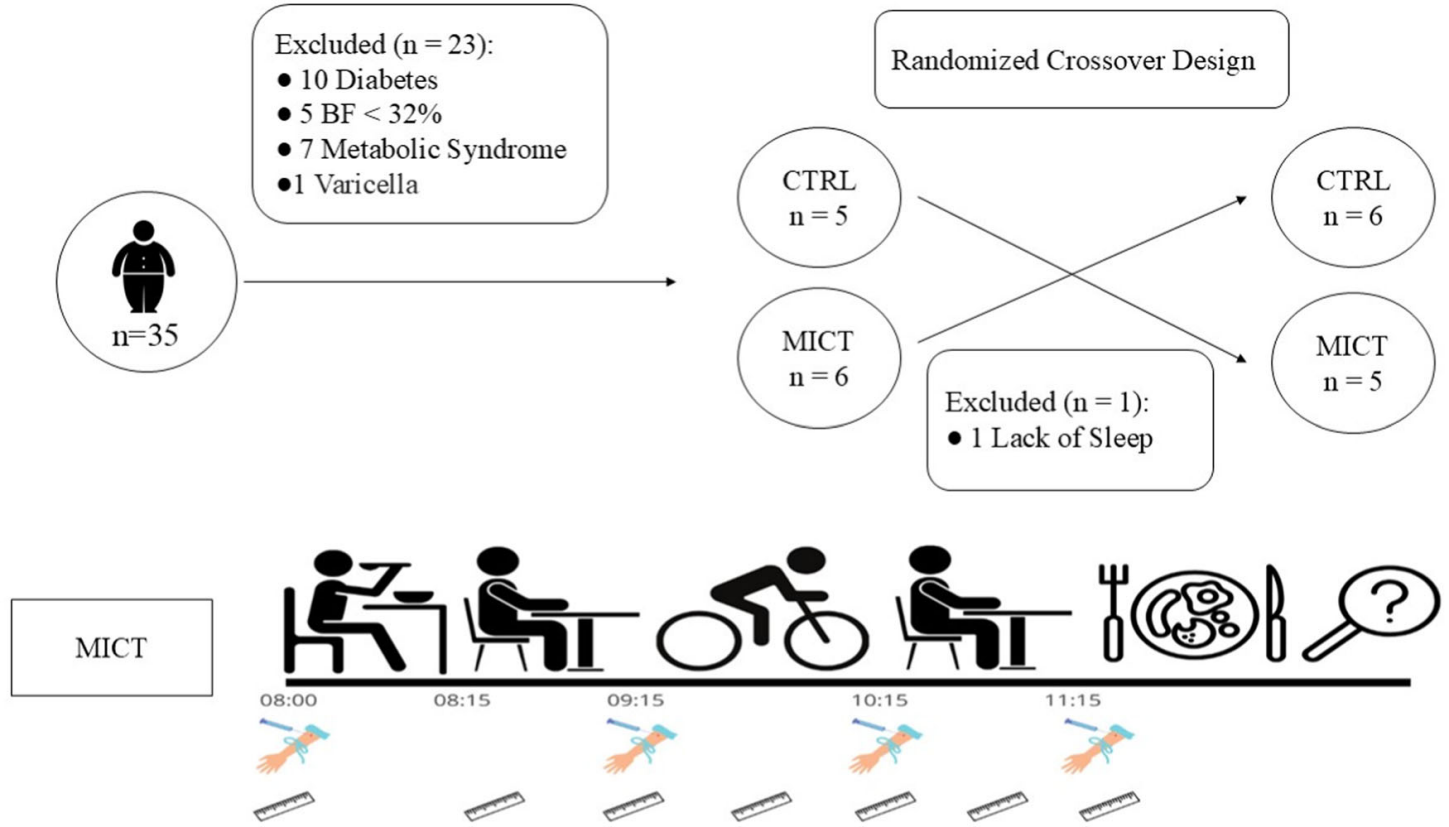
Selection flowchart of participants and the design of the study. Blood sampling time points are indicated by the hand while appetite perception is indicated by the ruler.

Participants screened into the study completed two 4‐h trials (one with exercise and one without) with at least 7‐days between sessions in a randomized crossover design. Participants were instructed to not consume alcohol and caffeine or take part in any exercise 48 h before the tests. Participants were asked to replicate food and beverage intake as closely as possible for the 24 h before each experimental session and were provided a standardized dinner on each of the evenings before the experimental sessions. Participants were asked not to consume anything but water after this meal and sleep at least 7 h. Upon arrival at the lab on the morning of the experimental sessions at 0800 h, participants were provided a standardized breakfast and given 1 h to rest. and digest before beginning the exercise session. At this point, participants either rested for 60 min or exercised at a moderate intensity (60%V̇O_2peak_) on a cycle ergometer for 60 min. Throughout the exercise session, energy expenditure was continuously measured breath by breath using a mask connected to gas analyzers, and heart rate was monitored (same as described above; Figure 1).

### 2.4 Subjective appetite perceptions

Appetite perceptions were assessed before breakfast, 30 min after breakfast, and in 30 min intervals until consumption of the ad libitum meal at 11:15 h using a 100 mm visual analog scale. The questions included hunger, satiation, fullness, and desire to eat.^19^ The overall appetite rating was calculated by taking the average of the four appetite ratings, with satisfaction and fullness scores being reverse‐scored.^20^

### 2.5 Blood sampling

All samples were collected at 0‐ (fasted), 75‐ (postprandial, pre‐exercise), 135‐ (0 min postexercise), and 195‐min (60 min postexercise) of each session via venipuncture from different points of the antecubital vein while participants remained seated. Venous blood samples were drawn into EDTA vacuum tubes (Xinle) then centrifuged at 1750*g* for 10 min at 4°C. Subsequently, the resulting supernatants were partitioned into storage tubes. To prevent ex vivo conversion of PYY_1–36_ to PYY_3–36_ DPP‐IV was added. These aliquots were then preserved at −80°C and later analyzed in batches upon study completion, aiming to minimize assay variability. PYY_3–36_ (ZB‐15208C‐H9648), total GLP‐1 (ZB‐10022C‐H9648), GDF‐15 (ZB‐10037C‐H9648), total ghrelin (ZB‐13091C‐H9648), and lactate (ZB‐LAC‐96A) were measured following the ZellBio ELISA kit instructions (Human ELISA Kits, ZellBio GmbH). Intra‐assay coefficient of variation (CV) for GLP‐1, PYY_3‐36_, total ghrelin, and GDF‐15 was CV <10%, with an interassay CV < 12%. Intra‐assay CV for Lactate was 2.9%, and interassay CV was 3.5% (CV(%) = (SD/MEAN). Insulin was determined by ELISA kits (5825‐300 A, Monobind), with an intra‐assay CV of 7.4%. Serum glucose, HDL, LDL, TG, and total cholesterol were measured using commercial kits (Pars Azemoon).

### 2.6 Food provision and ad libitum energy intake

Participants consumed a standardized dinner (Macaroni and beef), which provided 40% of their daily energy (4905 ± 408 kJ) requirement and contained 50% carbohydrate, 38% fat, and 12% protein. The standardized breakfast (white bread, strawberry jam, margarine, banana, and orange juice) provided 30% of their estimated daily energy needs based on the Mifflin equation.^21^ The mean breakfast energy was 3678 kJ, consisting of 71% carbohydrate, 19% fat, and 10% protein. Following the experimental session, participants had access to an ad libitum meal at 3:30 h, which included options like chicken‐salad sandwiches, kettle chips, chocolate cupcakes, bananas, apples, orange juice, and water. Participants were allowed 30 min to eat and were instructed to eat as much as they would like. Individuals ate their meals in isolation to eliminate social influence on eating habits,^22^ preventing any external factors from affecting their eating speed, food choices, or the amount consumed. The food and leftovers were weighed and manufacturer values were used to calculate the energy and macronutrient intakes (Table 1).

**Table 1:** Energy intake and macronutrient composition of the ad libitum meal.

	Control	Exercise
Energy intake (kJ)	5162.9 ± 1637.3	4674.5 ± 1545.9
Carbohydrate (g)	92.9 ± 28.4	80.8 ± 27.7
Fat (g)	54.4 ± 17.0	48.8 ± 16.3
Protein (g)	58.8 ± 18.5	55.8 ± 17.5

### 2.7 Sample size calculation

A sample size estimate was conducted using G *Power 3.1. Using an effect size of f = 0.5, which was based on the session x time difference (partial eta squared = 0.2) observed in PYY in a similar study design^23^ and with alpha set at 0.05 and power at 0.80, it was determined that a minimum of 11 participants would be required to detect significant differences.

### 2.8 Statistical analysis

Data were analyzed using SPSS software version 29 (IBM Corporation) for Windows. A repeated‐measures two‐way analysis of variance assessed variations between trials over time concerning appetite and parameters. Upon identifying significant main and interaction effects, post hoc analysis was conducted using Bonferroni correction to address multiple comparisons. An independent *t* test was implemented to compare all area under the curve (AUC) values as well as energy intake (kJ) in the control and experimental sessions. Statistical significance was accepted as *p *˂ 0.05. Effect sizes are provided to complement the results, including partial eta squared ($\eta_{p}^{2}$; small 0.01, medium 0.06, large 0.14) for main effects and interactions. The 95% confidence intervals (CI) were calculated for mean absolute pairwise differences between experimental trials and sessions. All data are presented in the figures as the mean ± SD.

## 3 RESULTS

Thirty‐five males expressed interest in participating in the study. However, only 12 individuals met the inclusion criteria, and one of these individuals was excluded during the second experimental session due to insufficient sleep (before data collection).

### 3.1 Exercise responses

The exercise session V̇O_2peak_: 28.4 ± 3.4 mL·kg^−1^·min^−1^ and the exercise heart rate was 142 ± 13 beats·min^−1^ (~72% of heart rate maximum) with a workload of 80 ± 30 W. The energy expenditure was recorded at 2724 ± 585 kJ.

### 3.2 GDF‐15

**Figure 2:**
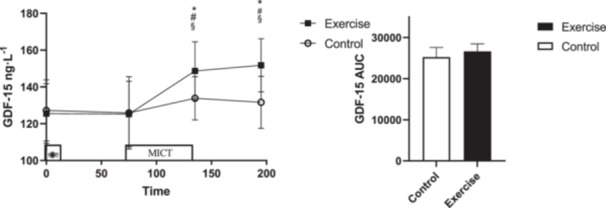
GDF‐15 concentrations across all time points during each experimental session. * denotes significantly different from control condition; § denotes significantly different from fasting; # denotes significantly different from pre‐exercise; $ denotes significantly different from fasting; # denotes significantly different from pre‐exercise; $ denotes significantly different from immediately after exercise. AUC for GDF‐15 across all time points during each experimental session. * donates significant changes.

An interaction (Time × Session) was observed for GDF‐15 (*p* = 0.007, $\eta_{p}^{2}=0.183$) where GDF‐15 was elevated immediately after exercise (95% CI: [2.48–27.28] ng/L, *p* = 0.021) and at 1 h postexercise (95% CI: [7.43–32.85] ng/L, *p* = 0.004) compared to CTRL. The concentrations GDF‐15 were also increased immediately postexercise compared to pre‐exercise (95% CI: [9.63–37.34] ng/L, *p* = 0.001) and fasting (95% CI: [4.75–41.60] ng/L, *p* = 0.009) and was also higher 1 h postexercise compared to pre‐exercise (95% CI: [11.03–42.10] ng/L, *p* = 0.001) and fasting (95% CI: [10.62–41.90] ng/L, *p* = 0.001). In addition, there was no difference for GDF‐15 AUC (*p *= 0.135, *d *= −0.66) (Figure 2).

### 3.3 Total ghrelin

**Figure 3:**
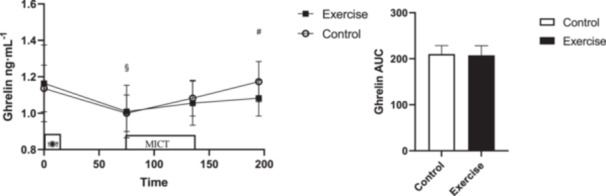
Total ghrelin concentrations across all time points during each experimental session. * denotes significantly different from control condition; § denotes significantly different from fasting; # denotes significantly different from pre‐exercise; $ denotes significantly different from immediately after exercise. AUC for total ghrelin across all time points during each experimental session. * donates significant changes.

There was no interaction (Time × Session) (*p* = 0.183, $\eta_{p}^{2}=0.077$) or main effect of session (*p* = 0.646, $\eta_{p}^{2}=0.011$) for total ghrelin, but there was a main effect of time (*p* < 0.001, $\eta_{p}^{2}=0.343$). The pairwise comparison indicated total ghrelin decreased immediately postexercise time compared to the fasting (95% CI: [(−0.22)–(−0.07)] ng/mL, *p* < 0.001) and increased in 1‐h postexercise compared to the pre‐exercise after the breakfast meal (95% CI: [0.05–0.19] ng/mL, *p* < 0.001). There was no difference in ghrelin AUC (*p *= 0.749, *d *= 0.14) (Figure 3).

### 3.4 PYY_3‐36_

**Figure 4:**
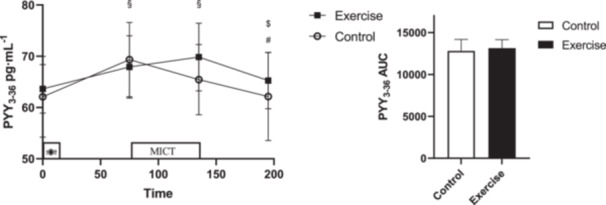
PYY3‐36 concentrations across all time points during each experimental session. * denotes significantly different from control condition; § denotes significantly different from fasting; # denotes significantly different from pre‐exercise; $ denotes significantly different from immediately after exercise. AUC for PYY3‐36 across all time points during each experimental session. * donates significant changes.

An interaction (Time × Session) was observed for PYY_3–36_ (*p* = 0.016, $\eta_{p}^{2}=0.158$) where PYY_3–36_ was increased pre‐exercise after the breakfast meal (95% CI: [0.95–7.58] pg/mL, *p* = 0.007) and immediately postexercise (95% CI: [1.70–10.69] pg/mL, *p* = 0.004) compared to fasting during moderate‐intensity cycling exercise (MICT). Although there was an increase between fasting and pre‐exercise (95% CI: [3.94–10.56] pg/mL, *p* = 0.001), there was no difference between immediately postexercise and fasting (95% CI: [(−1.15)–7.84] pg/mL, *p* = 0.25) during the CTRL session. The concentration of PYY_3–36_ decreased in both sessions after 1 h postexercise (control: 95% CI: [0.05–6.54] pg/mL, *p* = 0.04), (Exercise: 95% CI: [1.33–7.81] pg/mL, *p* = 0.003) compared to immediately postexercise. There was no difference (*p *= 0.542, *d *= −0.26) in PYY AUC (Figure 4).

### 3.5 Total GLP‐1

**Figure 5:**
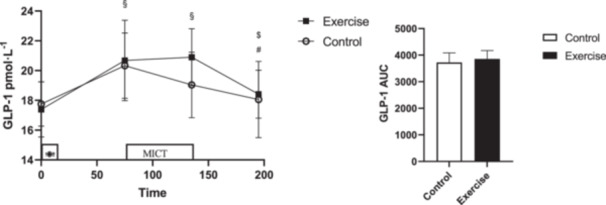
GLP‐1 concentrations across all time points during each experimental session. * denotes significantly different from control condition; § denotes significantly different from fasting; # denotes significantly different from pre‐exercise; $ denotes significantly different from immediately after exercise. AUC for GLP‐1 across all time points during each experimental session. * donates significant changes.

There was no interaction (Time × Session) (*p* = 0.088, $\eta_{p}^{2}=0.102$) or main effect of session (*p* = 0.451, $\eta_{p}^{2}=0.029$) for total GLP‐1. There was a main effect of time (*p* < 0.001, $\eta_{p}^{2}=0.490)$ where GLP‐1 increased pre‐exercise after the breakfast meal (95% CI: [1.52–4.34] pmol/L, *p* < 0.001) and remained elevated immediately postexercise (95% CI: [0.90–3.89] pmol/L, *p* = 0.001) compared to fasting condition. Total GLP‐1 were decreased at 1 h postexercise compared to pre‐exercise (95% CI: [(−3.47)–(−1.08)] pmol/L, *p* < 0.001) and compared to immediately postexercise (95% CI: [(−2.89)–(−0.58)] pmol/L, *p* = 0.002). There was no difference (*p *= 0.374, *d *= −0.39) in total GLP‐1 AUC (Figure 5).

### 3.6 Lactate

**Figure 6:**
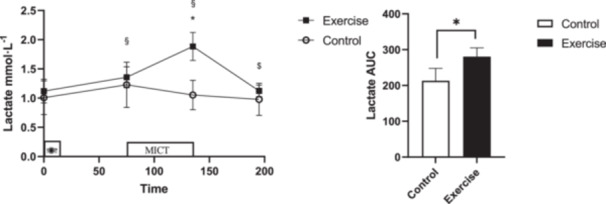
Lactate concentrations across all time points during each experimental session. * denotes significantly different from control condition; § denotes significantly different from fasting; # denotes significantly different from pre‐exercise; $ denotes significantly different from immediately after exercise. AUC for lactate across all time points during each experimental session. * donates significant changes.

An interaction (Time × Session) was observed for lactate (*p* < 0.001, $\eta_{p}^{2}=0.353$) where lactate was reduced at 1‐h postexercise compared to immediately postexercise (95% CI: [(−1.02)–(−0.50)] mmol/L, *p* < 0.001). Lactate was also increased immediately postexercise compared to pre‐exercise (95% CI: [0.25–0.79] mmol/L, *p *< 0.001) and fasting (95% CI; [0.41–1.11] mmol/L, *p* < 0.001) in MICT session and there were no significant difference in CTRL session in no time points. There was a difference (*p *< 0.001, *d *= −2.23) between MICT and CTRL for lactate AUC (Figure 6).

### 3.7 Insulin

There was no significant interaction (Time × Session) (*p* = 0.24, $\eta_{p}^{2}=0.067$) or main effect of session (*p* = 0.309, $\eta_{p}^{2}=0.052$) for insulin, but there was a main effect of time (*p* < 0.001, $\eta_{p}^{2}=0.213$) where insulin concentrations were increased pre‐exercise after breakfast consumption (95% CI: [50.39–107.64] µIU/mL, *p* < 0.001) and remained elevated immediately postexercise (95% CI: [1.25–41.45] µIU/mL, *p* = 0.033) compared to the control condition. There was no difference (*p* = 0.277, *d* = 0.47) in insulin AUC between conditions.

### 3.8 Glucose

There was no interaction (Time × Session) (*p* = 0.981, $\eta_{p}^{2}=0.001$) or main effect of session (*p* = 0.811, $\eta_{p}^{2}=0.003$) for glucose, though there was a main effect of time (*p* < 0.001, $\eta_{p}^{2}=0.558$) where glucose was higher at pre‐exercise compared to fasting (95% CI: [17.82–43.64] mg/dL, *p* < 0.001), immediately postexercise (95% CI: [13.79–48.84] mg/dL, *p* < 0.001), and 1 h postexercise (95% CI: [16.49–45.05] mg/dL, *p* < 0.001). There was no difference (*p* = 0.807, *d* = −0.10) in glucose AUC.

### 3.9 Subjective perception of appetite

**Figure 7:**
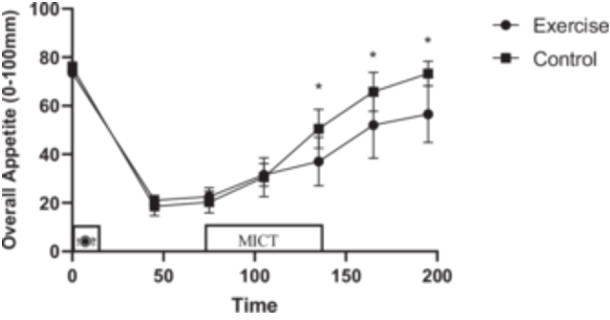
Overall appetite perceptions across all time points during each experimental session. * denotes significantly different from the control condition.

An interaction (Time × Session) was observed for overall appetite (*p* < 0.001, $\eta_{p}^{2}=0.339$) where overall appetite was decreased immediately postexercise (95% CI: [(−19.54)–(−3.40)] mm, *p* = 0.008) through 1‐h postexercise during MICT [95% CI (−22.38)–(−6.25)] mm, *p* = 0.001) compared to CTRL (Figure 7).

### 3.10 Energy intake

There was no difference in energy intake between sessions (95% CI: [(−1904.9)–928.1] kJ, *p* = 0.480) and no difference in macronutrient consumption between sessions for carbohydrate (95% CI: [(−37)–12.9] kJ, *p* = 0.32), fat (95% CI: [(−20.3)–9.3] kJ, *p* = 0.44), and protein (95% CI: [(−19)–13.1] kJ, *p* = 0.70).

## 4 DISCUSSION

Current research provides conflicting responses of gastrointestinal hormones to exercise in people with obesity, as classified by BMI.^6^ Notably, the present study is the first to use body composition as the primary criterion for participant selection. Our main findings confirm (i) GDF‐15 increases following moderate‐intensity aerobic exercise; (ii) subjective appetite was suppressed postexercise; and (iii) there were no significant changes in energy and macronutrient intake, total ghrelin, total GLP‐1, or PYY_3–36_ in response to exercise in individuals with obesity.

In line with previous studies on lean individuals,^10^, ^11^ the present results demonstrate an ~20% increase in GDF‐15 postexercise that remained elevated for at least an hour postexercise. Previous work has also demonstrated a 37% increase in GDF‐15 following a MICT (60‐min at 67% V̇O_2peak_) session.^11^ GDF‐15 receptors are present in specific brainstem regions, namely the area postrema and nucleus tractus solitarius, which are important areas in appetite control^24^ and increases in GDF‐15 concentrations are associated with a reduction in energy intake, suggesting an important role in the regulation of both appetite and body weight.^24^

While prior^18^, ^22^, ^25^, ^26^ studies have highlighted that total GLP‐1 concentration increases following a MICT session of varying durations (20–60 min) in individuals who experience overweight or obesity, we did not observe significant changes in males solely with obesity. Our study focused only on people with obesity and the results are in agreement with Bornath and colleagues, who did not see significant changes in active GLP‐1 after a 60 min MICT (65% V̇O_2max_) session in people with obesity.^23^ In contrast, Douglas and colleagues reported that a 60 min MICT session (60% V̇O_2peak_) increased total GLP‐1, while a study by Martins and colleagues demonstrated a similar increase following a MICT session (70% HR_max_) designed to elicit 250‐kcal expenditure (~45 min). Both studies included participants who were overweight or obese.^18^, ^22^ This is noteworthy because increases in total GLP‐1 have been seen following a 20‐min MICT session (70% HR_max_ or ~60% V̇O_2max_) in males with obesity.^26^ Therefore it appears that individuals who are overweight but not experiencing obesity may be driving the exercise response in those studies showing an increase in GLP‐1 following exercise. Further, the contrasting responses may have been driven by the significant increase at the pre‐exercise (postfeeding) time point (relative to fasting concentrations).

Increases in total PYY have been reported in individuals with overweight and obesity after 60 min of MICT (at 60% V̇O_2peak_) or 60 min at 50% V̇O_2max_.^22^, ^25^ However, another study^27^ did not see changes in total PYY after 30 min of exercise at 60% V̇O_2peak_ in people with overweight and obesity. Moreover, two studies investigated the effects of exercise on appetite control in people with obesity, and found neither 60 min of MICT at 65% V̇O_2max_ ^23^ or 60 min of MICT at 70% HR_max_ changed active PYY in people with obesity or overweight.^18^ Our findings indicate that active PYY concentration was not different between sessions, though it was acutely elevated postprandially. Previous findings indicate that longer duration MICT sessions (i.e., 60–90 min) are associated with higher total PYY concentrations in lean individuals,^13^, ^28^, ^29^, ^30^ while our study and a previous study indicate active PYY does not increase in response to MICT in individuals with obesity.^23^ Despite the initial postexercise increase in anorexigenic peptides, the concentrations of peptides tend to revert to pre‐exercise values within 1 h.^22^, ^31^ This pattern of reduction has been documented in prior studies, although the majority of these studies were carried out under conditions of overnight fasting. Interestingly, studies, where exercise was conducted after breakfast, demonstrated a reduction in PYY levels approximately 2 h after the exercise session,^30^ which contrasts with our own findings.

Matos and colleagues reported that 20 min of MICT at 70%HR_max_ reduced total ghrelin for up to 1 h postexercise in males with obesity.^32^ However, the present study observed no impact of MICT on total ghrelin concentration. Lactate has been implicated in the suppression of ghrelin secretion from gastric antrum P1/D1 cells posthigh‐intensity exercise.^8^, ^33^ The small increase in lactate concentration (~1 mmol/L) in response to the MICT may explain the lack of ghrelin suppression observed in the current study. Notably, Bornath and colleagues demonstrated a dysregulated acylated ghrelin response to MICT in individuals experiencing obesity compared to lean sedentary counterparts^23^ suggesting that the form of ghrelin measured is important.

In line with our study, Sim and colleagues reported that the plasma concentration of insulin did not change after exercise in people with obesity or overweight.^27^ MICT typically suppresses insulin concentrations due to the insulin‐like effects of exercise,^34^ allowing plasma glucose concentrations to be maintained with lower concentrations of insulin. Changes in insulin and glucose have been hypothesized to be involved in the appetite response to exercise though our data shows no effect on insulin and glucose.

In line with previous studies in people with overweight or obesity, appetite perceptions were suppressed following an acute session of MICT.^22^, ^26^ However, there is a contradictory result in males experiencing obesity.^23^ Despite these changes in appetite perceptions, the reduction in energy intake in the exercise compared to the CTRL session was not significantly different. A recent review has suggested that energy intake is reduced after acute exercise when participants had been fed.^35^ Although GDF‐15 increased significantly and appetite perceptions were suppressed, energy intake did not significantly reduce postexercise in males with obesity, but there was no compensatory energy compared to the CTRL session in spite of expending energy during MICT. In the current study, familiarizing participants with the food options during the preliminary session should have reduced the likelihood of overeating in the experimental sessions. However, the anticipation of receiving lunch may have psychologically affected their appetite.

The investigations of interactions between exercise and appetite‐regulating parameters remain relatively unclear, yet some mechanisms have been proposed.^7^ Recently, researchers have shed light on the role of certain myokines in influencing appetite (interleukin [IL]‐6, IL‐7),^9^ though recent evidence suggests that IL‐6 is not involved in exercise‐induced appetite suppression.^23^ Moreover, evidence indicates lactate may be a key player in exercise‐induced appetite suppression^33^ and recently it has been suggested that elevations in N‐lactoyl‐phenylalanine, a derivative of lactate and amino acid phenylalanine metabolism, generate the reduction in energy intake.^3^ A further mechanism that may be involved in the interplay to suppress appetite after exercise is increasing body temperature during exercise.^7^ More work is certainly warranted to improve our understanding of how exercise affects appetite‐regulating parameters.

To the best of our knowledge, this study was the first to select participants based on their body fat composition, while also measuring GDF‐15's role in appetite regulation following an acute session of MICT. While our study design is robust and the findings are important, the findings need to be interpreted in light of certain limitations. First, body fat measurement relied on bioelectric impedance, which while valid provides somewhat limited information. Considering the significance of fat distribution in appetite regulation, the study would have been strengthened with the use of dual‐energy x‐ray absorptiometry for body fat assessment. Another limitation was that only males were recruited for this study. Given the differing appetite responses between males and females,^36^ as well as altered body fat distribution between men and women, we suggest that future studies should be conducted with women living with obesity, selected based on their body fat levels. This could provide a more comprehensive understanding of total ghrelin and GLP‐1 at play in appetite regulation.

## 5 CONCLUSION

Our study indicates that GDF‐15 increases in individuals with obesity in response to a 60 min MICT exercise session which may have contributed to the suppression of subjective appetite. However, this MICT session did not affect other appetite parameters such as total ghrelin, total GLP‐1, or active PYY and did not exhibit energy compensation after exercise. This work is important as it focuses only on individuals experiencing obesity whereas many other studies include individuals who are overweight and obese and the current participants were also characterized based on body fat %, rather than just BMI. These findings suggest that moderate aerobic exercise can be a suitable remedy to create a negative energy balance driven by the energy expenditure of the session and the lack of difference in energy intake between the CTRL and MICT sessions.

## AUTHOR CONTRIBUTIONS

**Shahin Khodabandeh**: Conceptualization; data curation; formal analysis; investigation; methodology; software; writing—original draft; writing—review and editing. **Farhad Rahmani‐nia**: Conceptualization; methodology; supervision; validation; writing—review and editing. **Bahman Mirzaei**: Project administration; writing—review and editing. **Timothy J. Fairchild**: Formal analysis; methodology; software; writing—review and editing. **Tom J. Hazell**: Formal analysis; methodology; software; writing—review and editing.

## CONFLICT OF INTEREST STATEMENT

The authors declare no conflict of interest.

## ETHICS STATEMENT

This study received approval from the Sport Sciences Research Institute of Iran's Ethics Advisory Committee (Approval ID: IR.SSRC.REC.1402.116) prior to its initiation and was conducted under good clinical practice guidelines. All participants provided written informed consent to participate in this study.

## TRANSPARENCY STATEMENT

The lead author Farhad Rahmani‐nia affirms that this manuscript is an honest, accurate, and transparent account of the study being reported; that no important aspects of the study have been omitted; and that any discrepancies from the study as planned (and, if relevant, registered) have been explained.

## Data Availability

The data sets used and/or analyzed during the current study are available from the corresponding author on reasonable request.
